# Retraction: Erectile dysfunction drugs altered the activities of antioxidant enzymes, oxidative stress and the protein expressions of some cytochrome P_450_ isozymes involved in the steroidogenesis of steroid hormones

**DOI:** 10.1371/journal.pone.0347086

**Published:** 2026-04-13

**Authors:** The PLOS One Editors

Following the publication of this article [1], concerns were raised about the results presented in Fig 1, and the article’s compliance with PLOS’ editorial policies. Specifically,

- All β-actin panels in Fig 1 of this article [1], all β-actin panels in Figs 1 of [2] and [3, retracted in 4], and all β-actin panels in Figs 2 and 3 of [5, retracted in 6] appear to partially or fully overlap, despite being used to represent different experimental conditions or samples obtained from different animal species, calling into question the reliability of the associated quantified results.
- The Materials and methods section of this article states that the ethics committee of Alexandria University, Egypt, approved the design and the protocol of the experimental work, but the article does not report an ethics approval reference number.

The corresponding author provided some of the original blot images underlying Fig 1 and a copy of an IACUC approval document. Upon editorial review of these Fig 1 data, PLOS noted that the raw blot data for the β-actin panels of concern were not provided. Editorial assessment of the ethics approval document identified concerns about the integrity of this document, raising further concerns about the article’s compliance with the *PLOS One* Animal Research policy.

In light of these concerns, which call into question the integrity and reliability of the published results, the *PLOS One* Editors retract this article.

SAS and MEB did not agree with the retraction. AAM and MSS either did not respond directly or could not be reached.
